# Improving the surface properties of an UHMWPE shoulder implant with an atmospheric pressure plasma jet

**DOI:** 10.1038/s41598-018-22921-6

**Published:** 2018-03-16

**Authors:** S. Van Vrekhem, K. Vloebergh, M. Asadian, C. Vercruysse, H. Declercq, A. Van Tongel, L. De Wilde, N. De Geyter, R. Morent

**Affiliations:** 1 Research Unit Plasma Technology, Department of Applied Physics, Faculty of Engineering and Architecture, Ghent University, Sint-Pietersnieuwstraat 41, B-9000 Ghent, Belgium; 2 Tissue Engineering Group, Department of Basic Medical Sciences, Faculty of Medicine and Health Sciences, Ghent University, De Pintelaan 185 6B3, 9000 Ghent, Belgium; 3 Department of Orthopedic Surgery and Traumatology, Ghent University Hospital, De Pintelaan 185 13K12, 9000 Ghent, Belgium

## Abstract

Insufficient glenoid fixation is one of the main reasons for failure in total shoulder arthroplasty. This is predominantly caused by the inert nature of the ultra-high molecular weight polyethylene (UHMWPE) used in the glenoid component of the implant, which makes it difficult to adhesively bind to bone cement or bone. Previous studies have shown that this adhesion can be ameliorated by changing the surface chemistry using plasma technology. An atmospheric pressure plasma jet is used to treat UHMWPE substrates and to modify their surface chemistry. The modifications are investigated using several surface analysis techniques. The adhesion with bone cement is assessed using pull-out tests while osteoblast adhesion and proliferation is also tested making use of several cell viability assays. Additionally, the treated samples are put in simulated body fluid and the resulting calcium phosphate (CaP) deposition is evaluated as a measure of the *in vitro* bioactivity of the samples. The results show that the plasma modifications result in incorporation of oxygen in the surface, which leads to a significant improved adhesion to bone cement, an enhanced osteoblast proliferation and a more pronounced CaP deposition. The plasma-treated surfaces are therefore promising to act as a shoulder implant.

## Introduction

Anatomic total shoulder arthroplasty (TSA) consists of a humeral and glenoid component. The humeral component is usually made of a cobalt-chromium alloy or titanium and the most commonly used glenoid component is a concave socket made of ultra-high molecular weight polyethylene (UHMWPE). The golden standard for fixation of the glenoid component is by means of bone cement, although also uncemented press-fit fixation is often used^1^. However even with advances in surgical techniques and implant design, glenoid component loosening remains a common cause for failure in TSAs^2,3^. The cause of this failure can be multifactorial and is subdivided in two main categories: (i) incorrect placement of the component, which can lead to eccentric loading of the glenoid and (ii) insufficient component fixation due to sub-optimal adhesion between UHMWPE and bone cement or between UHMWPE and bone. This insufficient adhesion occurs due to the nature of the used material. In spite of its excellent chemical properties, UHMWPE is an inert and non-polar material, which makes it difficult to bind adhesively. Previous studies have aimed at and succeeded at increasing this adhesion by using plasma technology^4,5^. In these studies, plasma activation and plasma polymerization of methyl methacrylate (MMA) were performed in a dielectric barrier discharge (DBD)-reactor in order to alter the surface chemistry of UHMWPE samples resulting in an improved adhesion between bone cement and UHMWPE or in case of uncemented press-fit fixation an enhanced interaction between osteoblasts and UHMWPE. Most of the past and current research on plasma activation to improve the wettability and adhesion of polymer surfaces is performed at low or medium pressures^6–9^. Also the mentioned previous studies focused on medium pressure plasma activation of UHMWPE. This activation step was found to lead to the incorporation of oxygen-containing hydrophilic functional groups, which resulted in an increased surface wettability and a subsequent improved adhesion with bone cement or enhanced osteoblast cell proliferation. However, performing the plasma activation process at sub-atmospheric pressures leads to some disadvantages, as vacuum equipment is needed, which increases the cost, and the flexibility of the process in terms of what areas are treated, is low. One way of overcoming these disadvantages is by using an atmospheric pressure plasma jet (APPJ). Working at atmospheric pressure eliminates the need for vacuum equipment, while the jet design allows treating specific areas of the sample. Research in literature shows that a helium (He) or He/O_2_ APPJ has been used to modify the topography of UHMWPE and improve its wear performance^10^. Additionally, adhesive forces between the UHMWPE and a borosilicate sphere, used as model material for bone, were increased^11^. It is the goal of this manuscript to use plasma activation on UHMWPE with an argon APPJ and focus more on the resulting changes in surface chemistry and their effect on the bioactivity of the material. The induced surface modifications are studied in detail using X-ray photoelectron spectroscopy (XPS), water contact angle (WCA) goniometry and atomic force microscopy (AFM). Literature also shows that the effect of plasma activation is prone to ageing and doesn’t remain constant in time^12–17^: the induced chemical changes are often found to be partially reversible as time increases. Two main events have been attributed to this phenomenon: post-treatment chemical reactions and surface relaxation^18^. This ageing effect is therefore also assessed in this work by measuring WCA values and performing XPS-analysis.

Furthermore, specific tests are performed to assess the effect of the plasma treatment for the case of a cemented component and the case of an uncemented press-fit component. For the former case, the plasma treatment effect on the adhesion with bone cement is investigated using pull-out tests, while for the latter case the effect on the osteoblast cell proliferation is tested using (3-(4,5-dimethylthiazol-2-yl)-5-(3-carboxymethoxyphenyl)-2-(4-sulfophenyl)-2H-tetrazolium) (MTS) assays. Additionally, the bioactivity of uncemented samples is examined as the use of bone cement as fixation method has shown to adversely affect the underlying bone. This has triggered research into alternatives such as uncemented metal-backed components^19–21^ and uncemented polyethylene components^1,22^. It has been proposed in literature that one of the essential requirements for an artificial material to bond to living bone is the formation of bone-like apatite, which consists of calcium phosphates (CaP), on the surface after implantation^23^. This CaP formation can be replicated in a simulated body fluid (SBF). In other words, the *in vivo* bone bioactivity can be predicted from the apatite formation on its surface in SBF^24^. Therefore, the deposition of CaP is also studied in this work by immersing the samples in SBF.

## Materials and Methods

### Materials

For the characterization of the surface modification, UHMWPE-film, purchased from Goodfellow Cambridge Ltd. (England), with a thickness of 0.50 mm is used. Samples of 20 mm (x-direction) by 10 mm (y-direction) are cut out of the film. For the cell test, discs with a diameter of 15 mm are punched out of the UHMWPE-film, while for the CaP deposition square samples of 10 mm by 10 mm were used. In regard to the pull-out tests, a medical grade UHMWPE-plate (Chirulen 1020, Quadrant EPP Belgium) is used as these pull-out tests cannot be performed on the very thin UHMWPE-films. Pieces with dimensions 30 mm × 9 mm × 4 mm are mulled from the plate. The bone cement is obtained from Huge Dental Material Co. (China) and consists of an MMA liquid component and a PMMA resin. Upon adding the liquid component to the PMMA resin, a bone cement paste with a curing time of 20 to 30 min is formed. The argon (Alphagaz 1) used as working gas in the plasma jet is purchased from Air Liquide (Belgium).

### Experimental set-up

**Figure 1:**
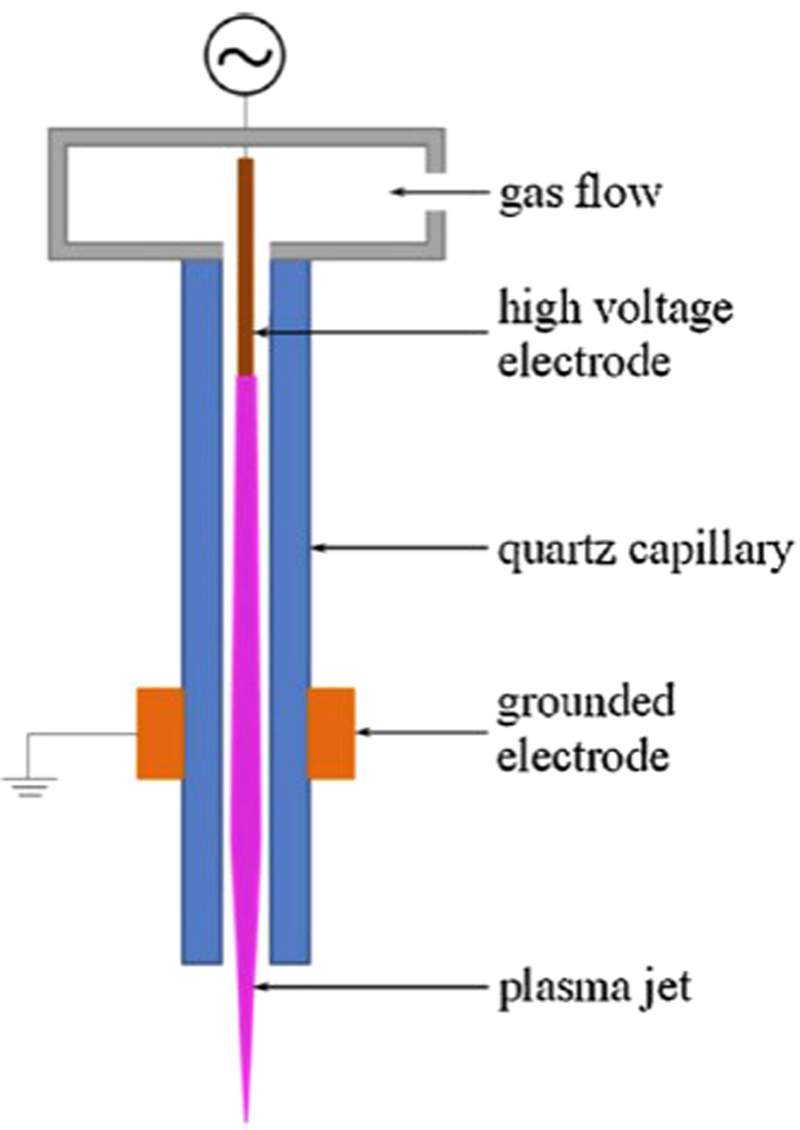
Schematic representation of the atmospheric pressure plasma jet configuration.

A schematic representation of the used atmospheric pressure plasma jet is displayed in Fig. 1. The device is made out of a quartz capillary with an inner diameter of 1.3 mm and an outer diameter of 3.0 mm and two electrodes. The needle electrode within the capillary is made of tungsten and is connected to a high voltage source (f = 27 kHz). Outside the capillary, there is a copper ring electrode with a height of 10 mm. The inter-electrode distance is fixed at 35 mm, while the distance between the center of the copper ring and the edge of the capillary is set at 20 mm. The nozzle-to-sample distance is fixed at 20 mm. An argon flow, controlled by a Bronckhorst EL-flow controller, going through the capillary is used as discharge gas. Depending on the experiments, the gas feed during plasma activation is set at 0.75 standard liters per minute (slm) or 2 slm while making use of a discharge power of 2.62 W and 1.60 W respectively. The plasma jet device is connected to a CNC portal milling machine (PF 600 P from BZT), which allows scanning the jet over the sample surface. The plasma jet moving velocity is set at either 50, 150 or 450 mm/min and repetitions range between 1 and 45. The resulting treatment time is calculated by dividing the diameter of the plasma afterglow by the jet velocity and multiplying this with the amount of repetitions. The created energy density is determined by multiplying the treatment time with the used discharge power, divided by the cross sectional area of the jet.

The plasma jet is programmed to scan the complete sample area using WINPC-NC software. As the plasma afterglow has a diameter of around 1 mm, the samples for surface characterization are scanned in 12 steps of 1 mm in the y-direction over a length of 22 mm in the x-direction. This is done to make sure the complete area is treated. In a similar way, the samples for the CaP deposition are scanned in 12 steps of 1 mm in the y-direction over a length of 12 mm in the x-direction. The samples for the cell tests are scanned in 17 steps of 1 mm in the y-direction over a length of 17 mm in the x-direction. For the samples for the pull-out tests, every face of the cuboid shape is plasma-treated and scanned analogously to the other samples, i.e. in such a way that the plasma jet surpassed every sample edge by 1 mm.

### Surface analysis techniques

To determine the effect of the plasma activation on the UHMWPE surface chemistry and morphology, several surface characterization techniques are performed. Static WCA measurements are obtained at room temperature to examine the effect of treatment time, argon gas flow and discharge power on the wettability of the polymer surface. These measurements are performed using a commercial Krüss Easy Drop system (Krüss Gmbh, Germany). Deionized water droplets of 1 μl are deposited on the surface, followed by automated measurements of the static contact angle by the instrument’s software. The obtained values are a result of Laplace-Young curve fitting and are the average of 5 measurements taken over an extended area of the sample.

Atomic force microscopy (AFM) is performed on three different locations per sample to assess the surface topography and roughness. 30 µm scans are recorded in a non-contact mode using an XE-10 AFM (Park Systems) with a silicon cantilever (Nanosensors™ PPP-NHCR) and XEP software is used to analyze the surface roughness.

X-ray photoelectron spectroscopy (XPS) measurements are done on a PHI 5000 Versaprobe II spectrometer employing a monochromatic Al K_α_ X-ray source (hν = 1486.6 eV) operating at 23.3 W. All measurements are conducted in a vacuum of at least 10^−6^ Pa and are taken at a take-off angle of 45° relative to the sample surface. Survey scans and individual high resolution C1s spectra are recorded with a pass energy of 187.85 eV and 23.5 eV respectively on 4 different point locations randomly selected on each sample. Elements present on the UHMWPE surfaces are identified from XPS survey scans and are quantified with Multipak software using a Shirley background and by applying the relative sensitivity factors supplied by the manufacturer. Multipak software is also used to curve fit the high resolution C1s peaks after the hydrocarbon component of the C1s spectrum (285.0 eV) was used to calibrate the energy scale.

The plasma ageing effect is assessed by resting the treated samples in ambient air at a fixed temperature of 20 °C and a fixed relative humidity of 50%, resembling controlled open air conditions. After five selected moments (4 h, 8 h, 24 h, 7 days and 14 days) the WCA is measured for each sample. Additionally, XPS-analysis is performed after an ageing time of 7 days.

### Pull-out tests

For the pull-out tests, medical grade UHMWPE samples are fixed in an MMA-based bone cement cylinder with a height of 40 mm and a diameter of 20 mm. The samples are fixed into the bone cement over a length of 10 mm. A sample holder was specially designed in order to be able to do this in a reproducible manner. Next, the samples are pulled out of the bone cement by a universal testing machine LRX plus (Lloyd Instruments, Bognor Regis, UK) with a fixed moving speed of 2 mm/min. For each condition, 10 samples are made and tested. A visualization of a similar set-up can be found in the work of Cools *et al*.^25^, which served as a basis for the pull-out tests in this study.

### Cell tests

The cell experiments consist of testing the cell viability of mouse calvaria 3T3 (MC3T3) sub clone pre-osteoblast cells on untreated and plasma-activated samples. Before culturing the cells, the samples are subjected to UV sterilization for 30 min. The cells are cultured with a density of 20000 cells per ml, with 1 ml per sample. After 24 h and after 7 days, cell viability is analyzed with a CellTiter 96® aqueous non-radioactive cell proliferation assay (Promega, USA). This MTS assay measures the cellular conversion of MTS into a soluble formazan dye by the mitochondrial NADH/NADPH-dependent dehydrogenase. The absorbance of the formazan dye in the solution is measured with a 490 nm Universal microplate reader EL 800 (BioTek Instruments, USA). The cell viability is calculated as a percentage of a control culture and each sample condition is tested in triplicate. Additionally, 7 days after cell seeding, a fluorescent live/dead staining with calcein AM (Anaspec, USA) and propidium iodide (Sigma Aldrich, Belgium) is performed, after which the live (green) and dead (red) cells are visualized with a fluorescence microscope (Olympus IX 81) making use of appropriate filters.

### CaP deposition

*In vitro* bioactivity of plasma-treated UHMWPE is investigated by immersion of the samples in 2.0 SBF for 14 days. Prior to the immersion, the samples are subjected to 6 cycles of alternate dipping. Each cycle consists of the following steps:

-
  - 60 s in 1000 mM Ca^2+^ (CaCl_2_∙2H_2_O; Merck 2382)
-
  - 30 s in H_2_O (for rinsing)
-
  - 60 s in 600 mM HPO_4_^2−^ (Na_2_HPO_4_.2H_2_O; Merck 6580)
-
  - 30 s in H_2_O (for rinsing)

2.0 SBF is prepared with the following components: NaCl (VWR Prolabo 27810.295), NaHCO_3_ (Merck 6329), KCl (Merck 4936), K_2_HPO_4_ (Merck 5104), MgCl_2_.6H_2_O (Merck 5833), CaCl_2_∙2H_2_O (Merck 2382), Na_2_SO_4_ (Merck 6647) and Tris(hydroxymethyl)aminomethane (VWR Prolabo 103156 × ). These components are dissolved in deionized water as described in the protocol of Kokubo *et al*.^26^ and the pH is adjusted to 7.4 with a 1 M HCl solution.

For each condition, 5 samples are immersed in SBF. After 14 days, FTIR spectra of the CaP-coatings are recorded using a Spectrum One spectrometer (Perkin Elmer Instruments, U.S.) for wavelengths between 4000 and 400 cm^−1^ with a resolution of 1 cm^−1^. Additionally, surface morphology analysis is performed using a JEOL JSM-6010 PLUS/LV scanning electron microscope (SEM). Prior to the SEM measurements, the samples are coated with a gold coating using a gold sputter coater (JFC-1300 autofine coater, JEOL, Japan). SEM images are acquired with an accelerating voltage of 7 kV at a working distance of 10 mm. Finally, optical micrographs are also recorded to obtain a comprehensive view of the homogeneity of the CaP deposition. All optical micrographs are recorded using a DZ 1100 stereomicroscope with a CMEX 5000 camera (Euromex). Image focus v3.0 is used to analyze the images.

## Results and Discussion

### Surface characterization

#### Water contact angle

**Figure 2:**
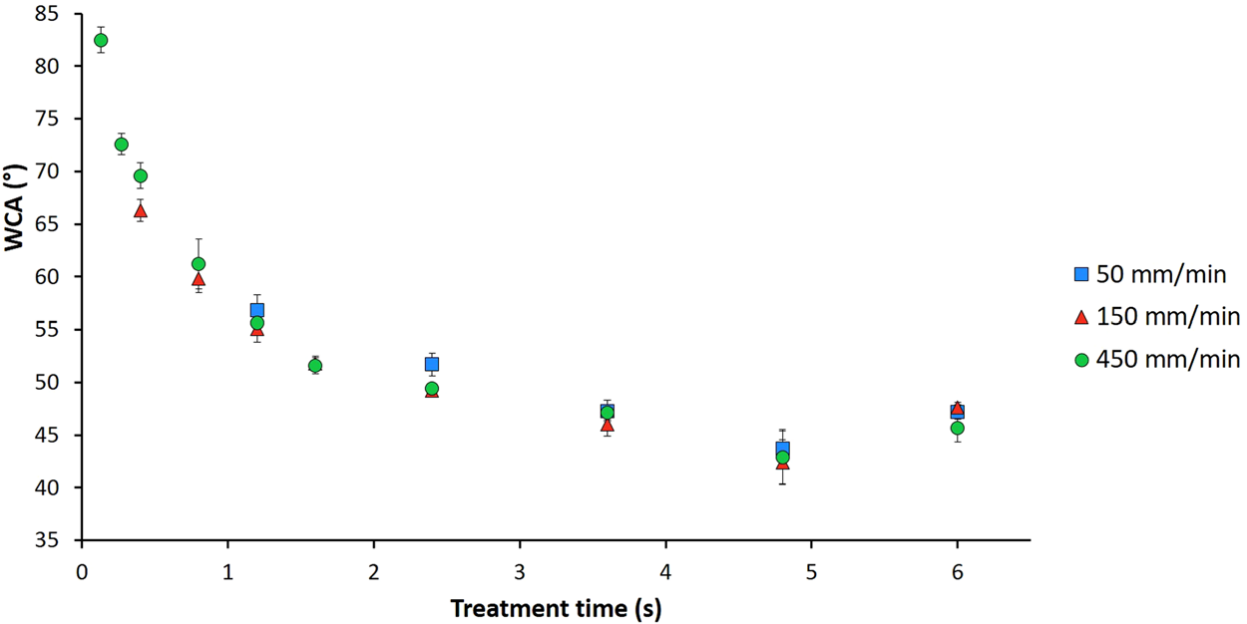
Water contact angle values as a function of treatment time for different jet velocities.

Figure 2 shows the results of the WCA measurements as a function of treatment time for different jet speeds and a fixed discharge power of 1.6 W. The WCA of untreated UHMWPE is 87° and decreases exponentially with increasing plasma treatment time for each of the 3 plasma jet velocities, until a plateau value of approximately 47° is reached. This plateau value is similar to a previously obtained value (49°) after Ar plasma treatment in a DBD-reactor at 5.0 kPa^4^. However, the energy density needed to obtain this plateau value in the DBD-reactor was 1.34 J/cm^2^ while the energy density needed with the plasma jet is calculated to be 564.14 J/cm². This difference in energy density can probably be explained by the manner of plasma exposure. In a DBD-reactor, the sample is in direct contact with the active plasma zone, while in the case of a plasma jet the sample is exposed indirectly to plasma as only the plasma afterglow reaches the surface. The decrease in WCA with treatment time can be attributed to the incorporation of oxygen-containing functional groups into the sample’s surface due to the treatment. This will be discussed in detail in the next section. Additionally, the results also show that the jet speed has no significant effect on the WCA, as using the same treatment time (by changing the number of repetitions) results in similar WCA values. For the same treatment time, using a higher jet speed with a high number of repetitions will therefore result in the same WCA value as using a lower jet speed with a low number of repetitions.

Based on these results, some specific plasma operational conditions were chosen to further investigate the plasma-induced effects on the surface chemistry, cell proliferation, adhesion to bone cement and CaP deposition. The chosen conditions are represented in Table 1 with their respective WCA values. Three different saturation conditions (S1 to S3) are first chosen to investigate whether the jet velocity has any effect at all. Condition B is a sample exposed to the plasma for a very short treatment time and is chosen to see the difference between saturated samples and samples that show a small treatment effect. Condition F is a combination of process parameters that is not represented in Fig. 2. The goal here is to see if an even more pronounced plasma treatment effect (high energy density resulting in a very low WCA value) has an influence on the results of the different tests. These parameters are based on previously obtained results^27^.

**Table 1:** Process parameters of selected conditions with their respective WCA and surface roughness values.

	Treatment time (s)	Jet velocity (mm/min)	Gas flow (slm)	Discharge power (W)	Energy density (J/cm²)	WCA (°)	Rq (nm)
Saturation 1 (S1)	4.68	450	2	1.60	564.14	47.1 ± 0.7	145 ± 22
Saturation 2 (S2)	4.68	150	2	1.60	564.14	46.0 ± 1.1	142 ± 34
Saturation 3 (S3)	4.68	50	2	1.60	564.14	47.3 ± 1.0	140 ± 27
Condition B	0.35	450	2	1.60	42.19	72.6 ± 1.0	124 ± 19
Condition F	4.68	50	0.75	2.60	916.73	25.2 ± 2.2	133 ± 27

#### XPS

The chemical composition of samples treated with the different process conditions are analyzed using XPS and compared with untreated samples. The results are presented in Table 2. The untreated UHMWPE samples did not contain any oxygen, however after plasma treatment the oxygen concentration is considerably increased. This explains the decrease in contact angle, as the incorporation of oxygen leads to a more hydrophilic surface. Conditions S1, S2 and S3 lead to a similar O/C-ratio of 0.43, showing that the jet velocity has no influence on the overall incorporated oxygen concentration. Using a lower treatment time (condition B) leads to a lower O/C-ratio. Although the WCA for condition F was significantly lower than for the other conditions, the measured O/C-ratio is similar to conditions S1, S2 and S3. This discrepancy can most likely be explained by the depth of analysis in the two techniques. WCA measurements analyze approximately the top 1 nm of the surface, while XPS analysis depends on the used take-off angle relative to the sample surface, which is 45° in this case and which results in an analyzing depth of approximately 10 nm. This seems to indicate that the oxygen concentration is not homogeneous over these 10 nm and that there probably is a gradual decrease of the oxygen content from the surface into the sample. Comparing these results to the O/C-ratio (0.27) obtained at medium pressure^4^, it is clear that condition B leads to similar results while the saturation conditions lead to significantly higher oxygen concentrations, which could not be reached making use of the medium pressure DBD.

**Table 2:** O/C-ratio and relative percentage of carbon-containing functional groups present on the sample’s surface for each condition.

	O/C	C-C/C-H (%)	C-O (%)	C = O (%)	O-C = O (%)
S1	0.43 ± 0.03	63.2 ± 2.4	14.2 ± 3.2	10.0 ± 2.1	12.6 ± 1.2
S2	0.41 ± 0.03	62.3 ± 4.9	17.2 ± 5.8	8.0 ± 1.5	12.5 ± 1.0
S3	0.40 ± 0.02	65.9 ± 6.4	18.5 ± 7.8	6.4 ± 1.6	9.1 ± 1.2
B	0.26 ± 0.03	72.6 ± 3.1	14.0 ± 3.5	8.4 ± 0.9	5.0 ± 0.6
F	0.42 ± 0.02	62.0 ± 3.7	20.5 ± 5.4	8.0 ± 2.2	9.5 ± 0.8

To investigate the chemical groups present on the treated samples, curve fitting of detailed C1s peaks is performed. Based on literature, the obtained C1s envelopes can be decomposed into 4 distinct components: a peak at 285.0 eV corresponding to C-C/C-H bonds, a peak at 286.5 eV attributed to C-O bonds present in alcohols and esters, a peak at 287.6 eV due to C = O bonds in aldehydes and ketones and a peak at 288.9 eV corresponding to O = C-O bonds in carboxylic acids and esters^28^. The results in Table 2 show that in terms of the type of incorporated oxygen-containing functional groups, the jet velocity does have an effect. Increasing the jet velocity seems to lead to higher carbonyl and carboxyl concentrations and a lower amount of C-O functionalities. This corresponds with other results found in literature^29^. Carton *et al*. hypothesized that the lower retention of carboxyls and carbonyls at lower jet velocities might be due to heating of the surface, which causes some of the carboxylic groups to be converted into CO_2_ or other volatile compounds. Table 2 also reveals that condition B leads to a lower amount of oxygen-containing functionalities, especially less carboxylic moieties. The relative percentages of oxygen-containing functional groups for condition F are quite similar to condition S3, showing that increasing the discharge power (i.e. energy density) does not have a significant effect on the way oxygen is incorporated into the surface.

#### AFM

The surface morphology is also researched using AFM and the acquired surface roughness (Rq) values for the different conditions are presented in Table 1. No significant plasma-induced effect on the surface roughness is found for all examined conditions, as the root mean square values for the treated samples are in the same order as for the untreated UHMWPE (R_q_ = 139 ± 8 nm). This corresponds to literature as it has already been reported that Ar plasma enhances radical reactions and restrains electron and ion etching effects^30^.

### Ageing

#### Contact angle

**Figure 3:**
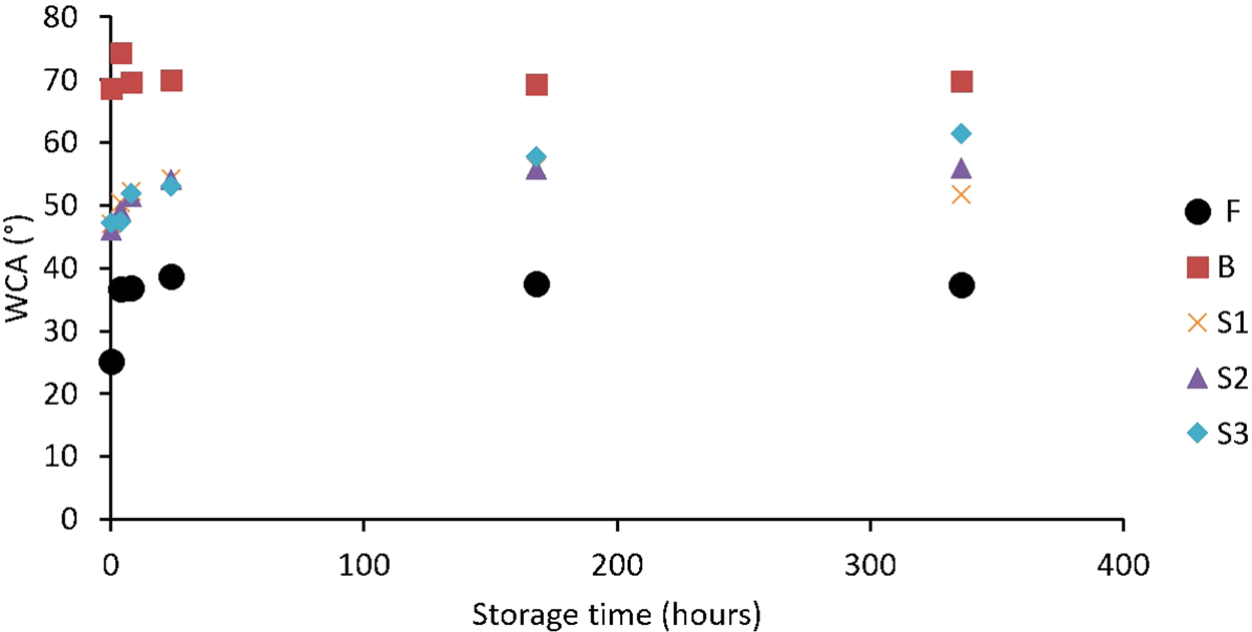
WCA values as a function of storage time after plasma treatment.

Figure 3 shows the evolution of the WCA as a function of storage time after plasma treatment. It is clear that for most samples the ageing effect stabilizes after 7 days of storage in ambient air. As ageing results in a loss of the treatment effect, the remaining treatment efficiency R (%) can be calculated using the following equation^31^:

1 $$R=100\cdot (\frac{{\theta }_{ageing}-{\theta }_{untreated}}{{\theta }_{sat}-{\theta }_{untreated}})$$

where θ_sat_ is the saturation value of the WCA after plasma treatment, θ_ageing_ is the value after 7 days of storage and θ_untreated_ is the WCA value of the untreated material, which is equal to 87° in this case. These calculated R values are presented in Table 3. As could be deduced from the WCA results, samples treated with condition B show little to no ageing and keep most of their plasma treatment effect as an R value equal to 96% is found in this case. After 7 days in ambient air, the remaining treatment effect for condition F is lower (80%). For the S1, S2 and S3 conditions, this remaining treatment effect is even lower and drops to 77%, 76% and 73% respectively. Overall, the preservation of the treatment effect is much more pronounced after treatment with the plasma jet than with a medium pressure DBD-reactor, where R values of only 50% were found^32^, which is an additional advantage of the APPJ.

**Table 3:** Percentage of remaining surface modification R, O/C-ratio and relative percentage of carbon-containing functional groups present on the surface after 7 days of ageing in ambient air.

	R (%)	O/C	C-C/C-H (%)	C-O (%)	C = O (%)	O-C = O (%)
S1	77	0.27 ± 0.01	73.1 ± 0.2	11.9 ± 0.2	8.2 ± 0.3	6.9 ± 0.4
S2	76	0.23 ± 0.03	72.6 ± 0.5	12.7 ± 0.1	8.1 ± 0.1	6.7 ± 0.5
S3	73	0.23 ± 0.02	74.1 ± 0.8	12.0 ± 0.5	7.5 ± 0.4	6.4 ± 0.5
B	96	0.12 ± 0.02	79.4 ± 0.8	12.4 ± 0.4	5.9 ± 0.5	2.3 ± 0.1
F	80	0.23 ± 0.04	73.3 ± 0.4	11.7 ± 0.1	7.6 ± 0.4	7.5 ± 0.3

#### XPS

Table 3 shows the influence of the ageing effect on the chemical composition of the plasma-activated samples after storage for 7 days. For all conditions, a significant decrease (40–50%) of the O/C-ratio is observed, which correlates with the increasing WCA values. Comparing the percentages of the different carbon-containing functional groups immediately after treatment and after a storage time of 7 days shows that the amount of C-C/C-H bonds has increased and the percentage of C-O and O = C-O bonds has decreased for all conditions, while the amount of C = O bonds has remained more or less the same. These XPS results thus indicate a reordening of the functional groups during the ageing process and can be attributed to the tendency of a surface to restore its original surface energy^18^.

### Adhesion tests

**Figure 4:**
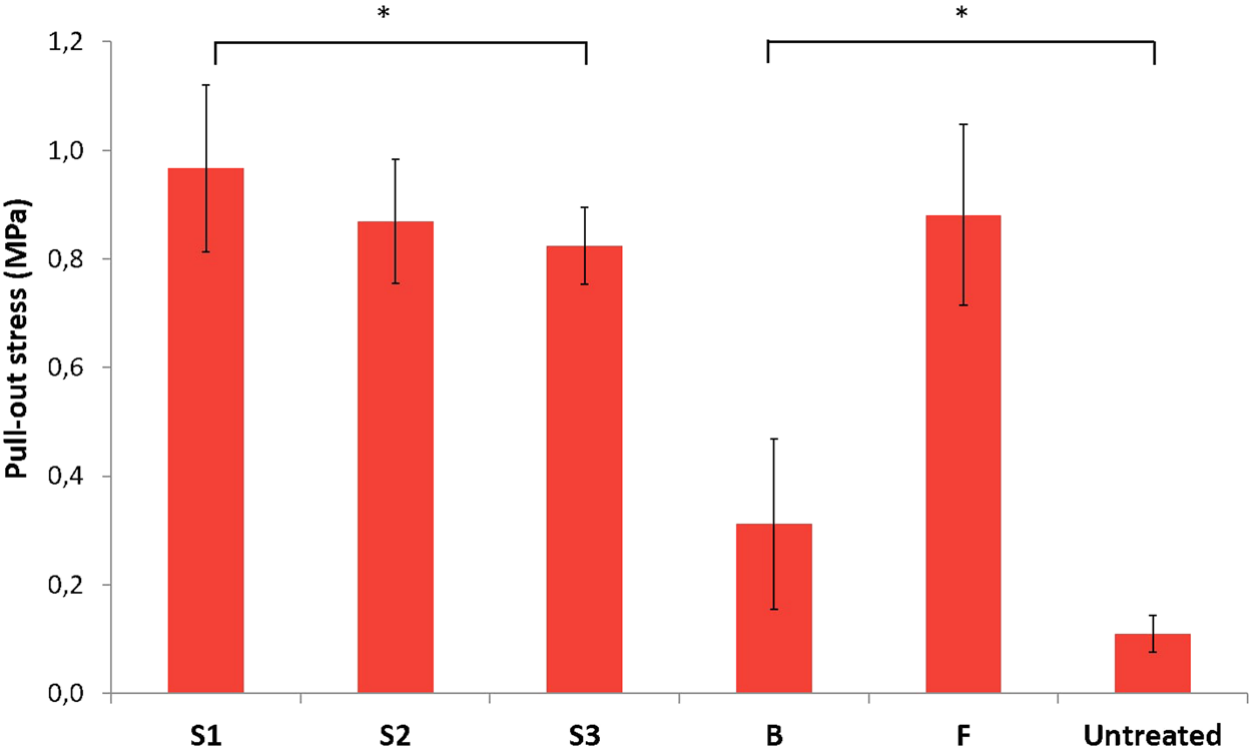
Pull-out stress for different process conditions.

In order to assess the effect of the plasma treatment on the adhesion between MMA-based bone cement and UHMWPE, samples are fixed in bone cement and subsequently pulled out by a universal testing machine. The force that is needed to remove the UHMWPE samples out of the bone cement can be used as a measure for the adhesion between the two components. Figure 4 shows the results of the pull-out tests depicting the pull-out stress for different samples. It is clear that all plasma treatment conditions lead to a significantly improved adhesion between UHMWPE and bone cement. Even condition B, which caused a relatively minimal surface modification, results in a statistically significant difference (p < 0.05) in pull-out stress. This increase in adhesion can be correlated with the increase in oxygen concentration on the sample’s surface. More oxygen-containing functional groups result in more interaction with the functional groups present in the bone cement and therefore lead to a stronger adhesion. This can be seen in the difference between samples treated with condition B and the other conditions (p < 0.01), as all the other conditions result in a significantly higher oxygen concentration and as a result a significantly higher pull-out stress. Additionally, the type of oxygen functionality also seems to have an effect on the pull-out stress as there is a statistically significant difference between the samples S1 and S3 but no significant difference in O/C-ratio. The XPS-results in Table 2 however revealed that the S1 samples have a higher concentration of carboxyls. As the bone cement is based on MMA, it will contain a significant amount of ester functionalities. Therefore, the more carboxylic functionalities are present on the treated samples, the stronger the Van der Waals interactions and subsequently the stronger the adhesion between the two components will be. Hence, based on these results it can be concluded that plasma activation of UHMWPE with an APPJ results in a better adhesion between bone cement and UHMWPE, and that it is beneficial to use higher jet velocities during plasma activation as it leads to the incorporation of a higher amount of carboxylic groups.

### Cell tests

**Figure 5:**
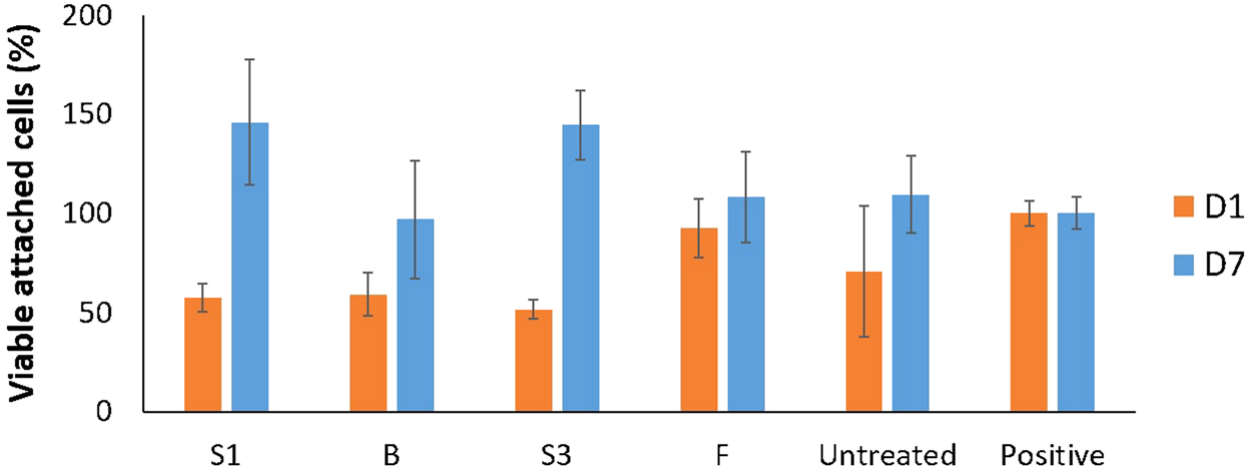
Percentage of viable attached MC3T3 cells for different conditions compared to a positive control culture for a culture time of 1 day and 7 days.

**Figure 6:**
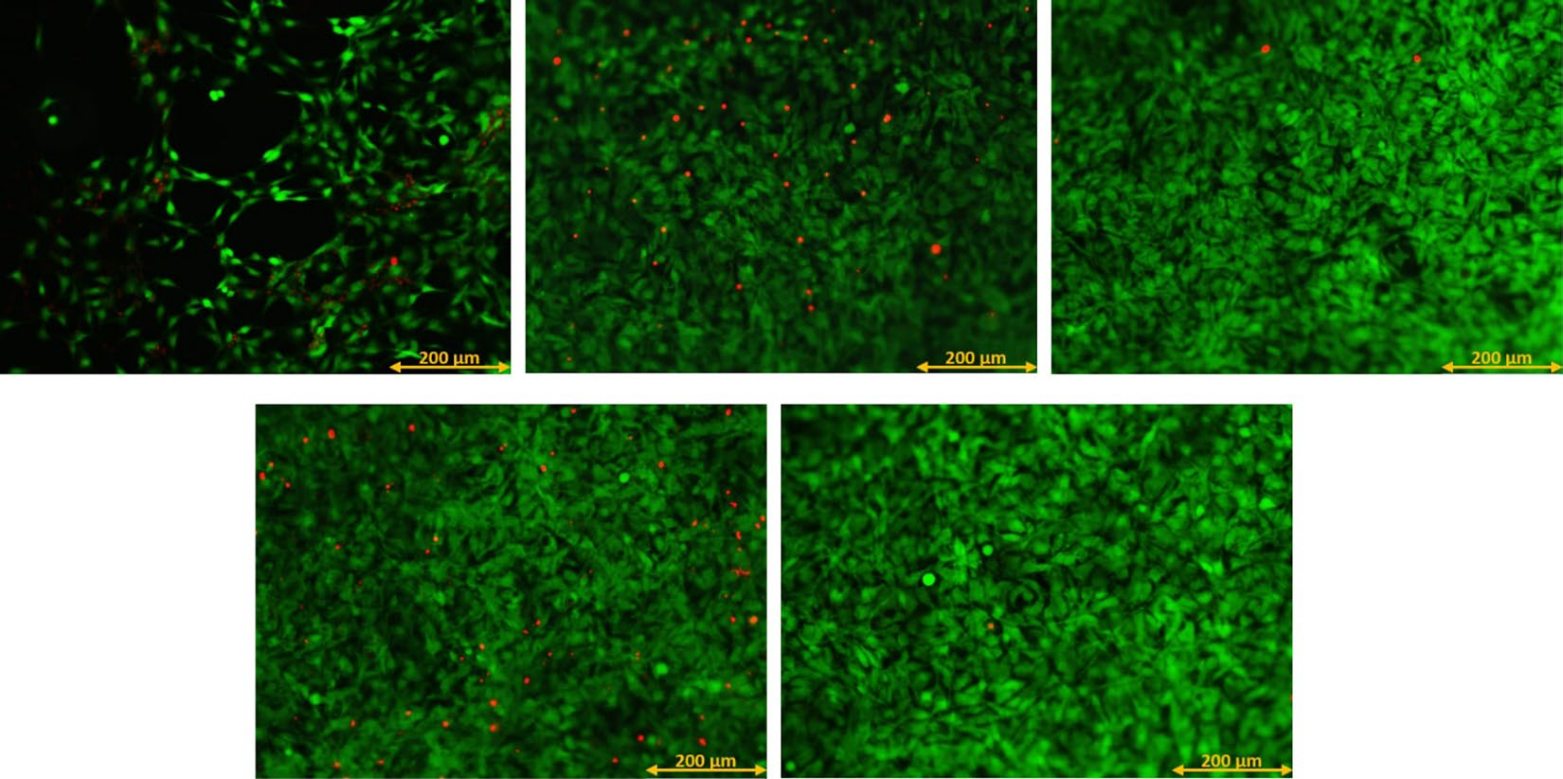
Fluorescence images of MC3T3 cells for different conditions after a culture time of 7 days. Upper row: Untreated (left), condition S1 (middle), condition S3 (right). Lower row: condition F (left), condition B (right).

Cell tests can be used to assess the viability of cells on a surface for different treatment conditions. Research has shown that multiple factors such as surface chemistry, topography and wettability influence the proliferation of cells on a substrate^33–38^. Earlier results have already showed that DBD-plasma treatment can significantly improve the proliferation of MC3T3 osteoblast cells on UHMWPE substrates^5^. Figure 5 shows the percentage of viable attached MC3T3 cells for different treatment conditions compared to a positive control culture for a culture time of 1 day and 7 days. These MTS assay results seem to indicate that (1) the S1 and S3 conditions lead to a significantly higher MC3T3 proliferation compared to the untreated sample 7 days after culturing and (2) the F and B conditions lead to a similar MC3T3 proliferation as the untreated sample 7 days after culturing. However, the fluorescence images depicted in Fig. 6 show that there is a significant difference in MC3T3 proliferation between the untreated sample on the one hand and all plasma-treated samples on the other hand. Additionally, there seems to be no large differences in MC3T3 proliferation amongst the different treatment conditions. The discrepancy between the fluorescence images and the results of the MTS assay can most likely be attributed to the low sensitivity of the MTS assay for this specific cell type. In this work, it can thus be stated that the conducted plasma treatments have a clear and significant effect on osteoblast cell viability, which can be attributed to the incorporation of oxygen in the surface and the subsequent change in surface wettability^5,33–37^. Furthermore, the fluorescence images shown in Fig. 6 demonstrate a difference in cell morphology between untreated and plasma-treated samples. While most of the cells on the treated samples have an elongated or triangular shape, which indicates attachment to the surface, the untreated samples contain considerably more cells with a round shape, which means these cells are not well attached to the surface. Therefore, it can be stated that plasma treatment not only improves MC3T3 proliferation, it also greatly enhances cell morphology and consequently cell adhesion on UHMWPE samples.

### CaP deposition

**Figure 7:**
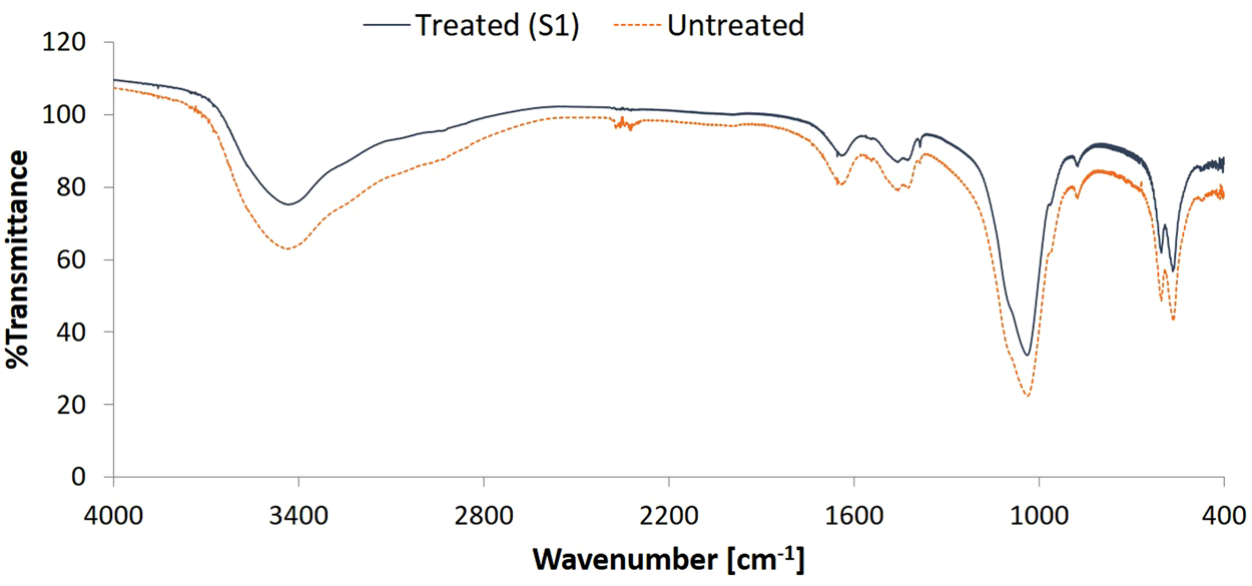
FTIR-spectra of the CaP-layer on plasma-treated (S1) and untreated samples after immersion in 2.0 SBF for 14 days.

**Figure 8:**
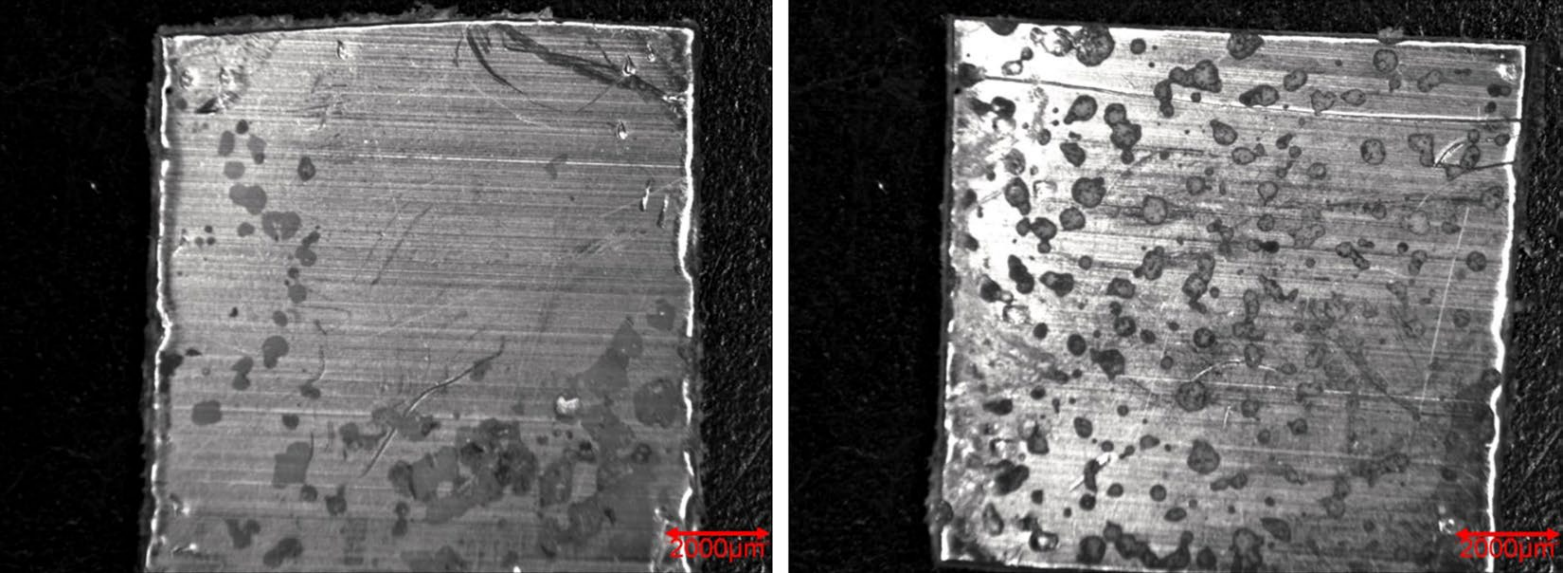
Micrograph of the CaP deposition on untreated (left) and plasma-treated (S1, right) samples.

**Figure 9:**
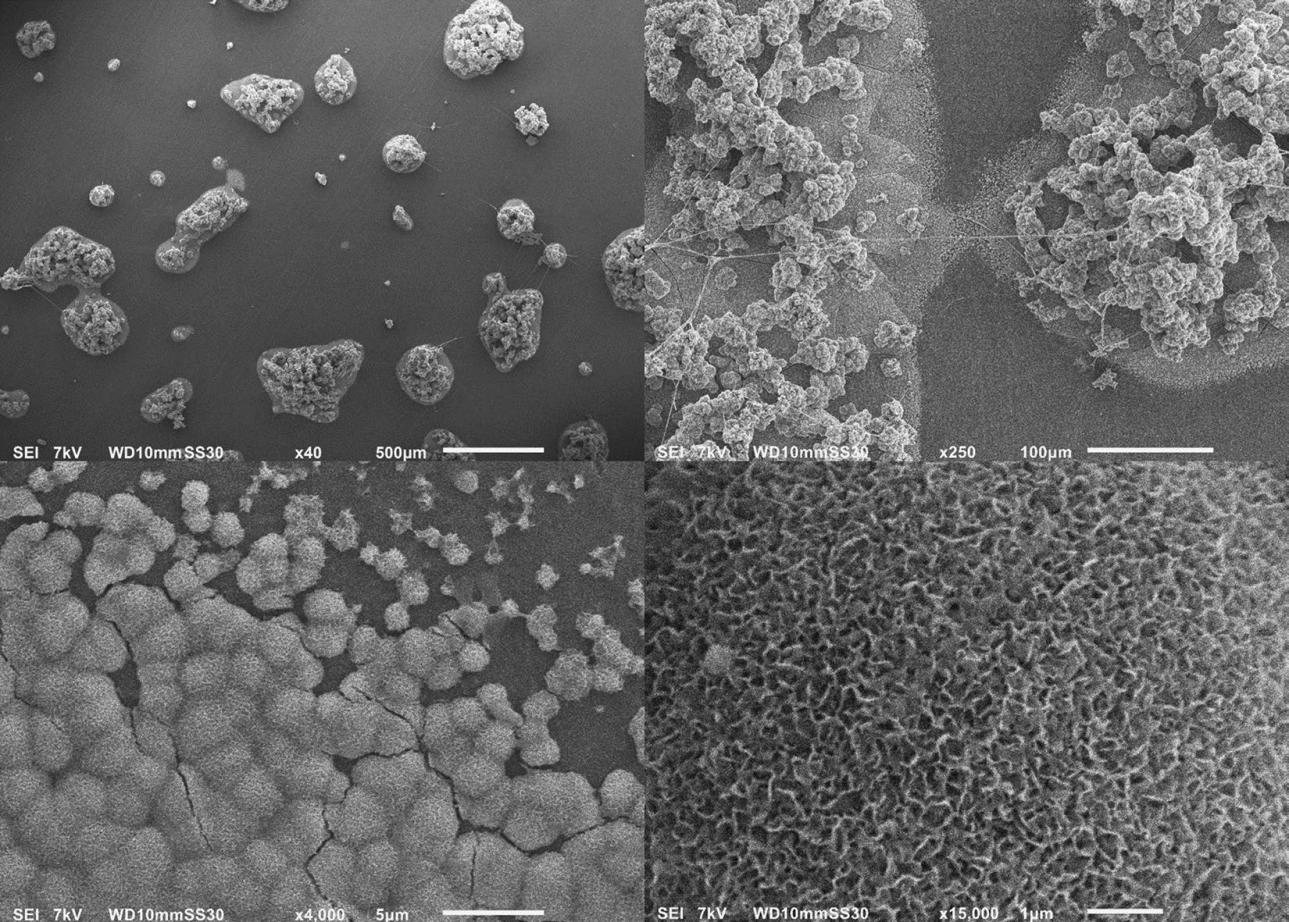
SEM images of plasma-treated samples (condition S1) for x40 (upper left), x250 (upper right), x4000 (lower left) and x15000 (lower right) magnifications.

**Figure 10:**
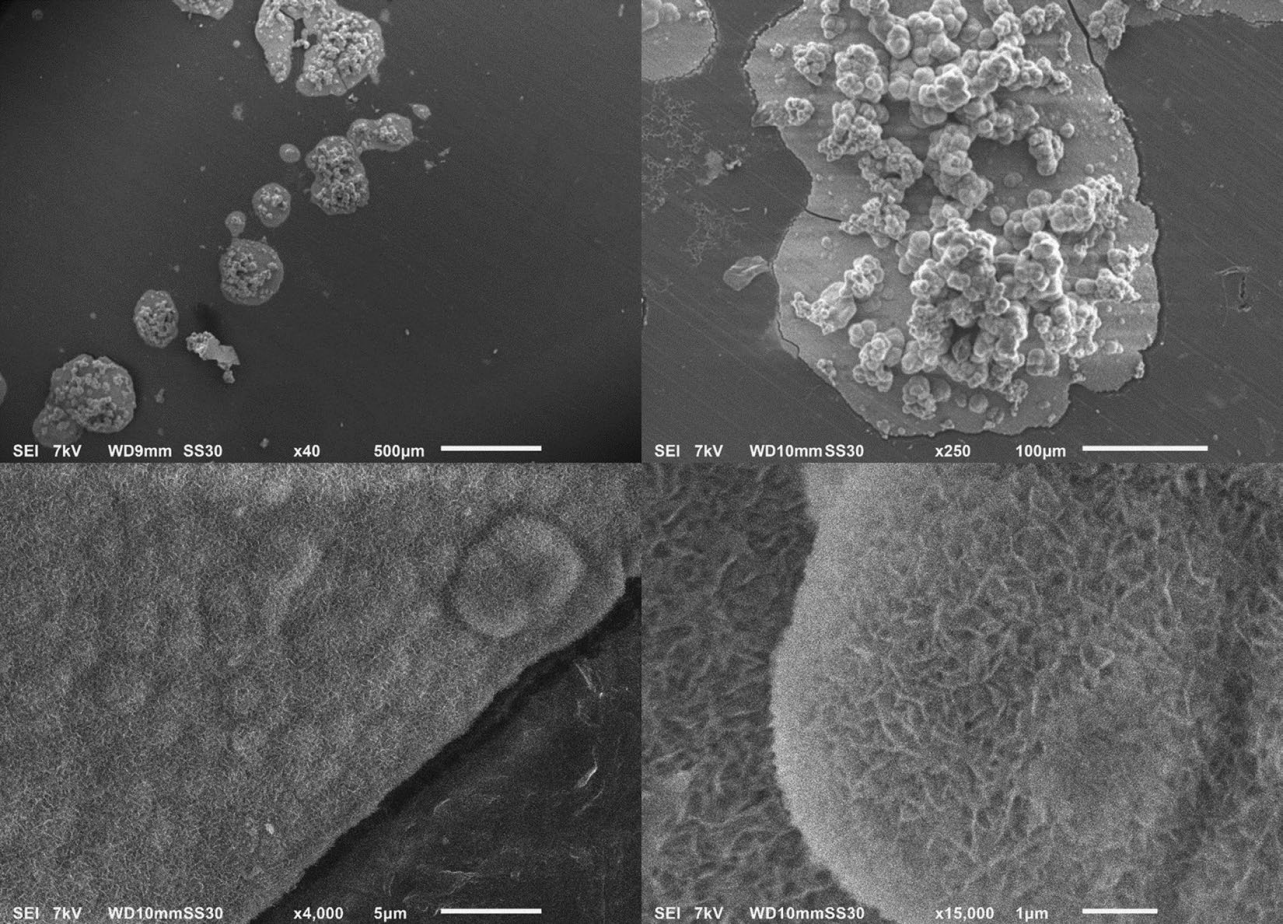
SEM images of untreated samples for x40 (upper left), x250 (upper right), x4000 (lower left) and x15000 (lower right) magnifications.

As mentioned before, the bioactivity of an uncemented sample can be examined by immersing it in SBF and studying the CaP formation on its surface. Figure 7 shows the FTIR-spectra of treated (condition S1) and untreated samples after immersion in 2.0 SBF for 14 days. It is clear that there is no significant difference between both spectra and therefore no difference in chemical composition of the CaP layer deposited on both sample types. The absorption bands at 560 cm^−1^, 600 cm^−1^ and around 1050 cm^−1^ show the presence of PO_4_^3−^ groups^39^. The absorption band from 2600 cm^−1^ to 3600 cm^−1^ can be attributed to absorbed H_2_O^40^. The absorption bands at 875 cm^−1^, 1420 cm^−1^, 1450 cm^−1^ and 1650 cm^−1^ arise due to the presence of CO_3_^2−^ groups, which substitute the phosphate ions^40,41^. It can therefore be concluded that B-type apatite is formed on both the untreated and plasma-treated samples^40^. Although there is no apparent difference in chemical composition, there is a significant difference in the homogeneity of the CaP layer on the different samples. As can be seen in the micrograph shown in Fig. 8, the CaP deposition does not cover the entire surface for both samples but is limited to different ‘islands’. On untreated UHMWPE however the deposition is localized near the edges of the sample, while on the plasma-treated sample the islands are distributed homogeneously over the entire surface. The homogeneity of the CaP deposition on the plasma-treated sample can be explained by the activation energy barrier that must be exceeded for nucleation to occur^42^. The homogeneous formation of functional groups such as –COOH and -COH on the surface due to the plasma treatment results in a decrease of the activation energy as the surface is saturated with nucleation sites. As such, a more homogeneous CaP deposition can be obtained on the plasma-treated sample. The SEM-images shown in Figs 9 and 10 confirm the formation of the CaP-islands and the obtained deposition seems to have a cauliflower shape with leaf-like shaped crystals for both samples. In conclusion, it can be stated that plasma treatment enhances the nucleation of a CaP layer on UHMWPE samples compared to untreated samples.

## Conclusions

The results of this paper show a promising step towards the use of an Ar APPJ for the surface modification of UHMWPE used in the glenoid component of shoulder implants. XPS analysis confirms the incorporation of oxygen into the surface and shows that increasing the jet velocity leads to higher carbonyl and carboxyl concentrations. As a result of this oxygen incorporation, the surface wettability also strongly increases. AFM analysis shows that the use of an Ar APPJ has no significant effect on the surface roughness. Pull-out tests reveal that the induced surface modifications significantly increase the adhesion between UHMWPE substrates and PMMA-based bone cement. Additionally, the pull-out stress depends on the concentration of carbonyl and carboxyl groups incorporated on the substrate’s surface: a higher carbonyl and carboxyl group content results in higher adhesion forces between the substrate and the bone cement. MTS cell proliferation assays and fluorescence images also confirm the enhancement of osteoblast viability and morphology on uncemented UHMWPE substrates due to the plasma-induced oxygen incorporation. Finally, analysis of the CaP formation after immersion of UHMWPE in 2.0 SBF shows that the bioactivity of the substrate is significantly enhanced as the homogeneity of the CaP deposition is improved due to the formation of functional groups on the surface. It can therefore be concluded that atmospheric pressure plasma treatment of UHMWPE may assist in resolving the problem of the loosening of both cemented and uncemented press-fit glenoid components in shoulder implants.
